# VExUS Protocol Along Cardiorenal Syndrome: An Updated Review

**DOI:** 10.3390/jcm14041334

**Published:** 2025-02-17

**Authors:** Amelia Campos-Sáenz de Santamaría, Zoila Stany Albines Fiestas, Silvia Crespo-Aznarez, Laura Karla Esterellas-Sánchez, Marta Sánchez-Marteles, Vanesa Garcés-Horna, Claudia Josa-Laorden, Alejandro Alcaine-Otín, Ignacio Gimenez-Lopez, Jorge Rubio-Gracia

**Affiliations:** 1 Internal Medicine Department, Hospital Clínico Lozano Blesa, 50009 Zaragoza, Spain or acampossa@salud.aragon.es (A.C.-S.d.S.); screspo@salud.aragon.es (S.C.-A.); lkesterellas@salud.aragon.es (L.K.E.-S.); msanchezmar@salud.aragon.es (M.S.-M.); vgarces@salud.aragon.es (V.G.-H.); jrubiogra@posta.unizar.es (J.R.-G.); 2 Aragon Health Research Institute (IIS Aragon), 50009 Zaragoza, Spain; zsalbines@salud.aragon.es (Z.S.A.F.); cjosal@salud.aragon.es (C.J.-L.); 3 Nephrology Department, Hospital Clínico Lozano Blesa, 50009 Zaragoza, Spain; 4 School of Medicine, University of Zaragoza, 50009 Zaragoza, Spain; 5 Computing for Medical and Biological Applications Group, Faculty of Health Sciences, University San Jorge, 50830 Villanueva de Gállego, Spain; lalcaine@usj.es; 6 Biomedical Research Center of Aragon (CIBA), 50009 Zaragoza, Spain

**Keywords:** acute kidney injury, cardiorenal syndrome, chronic kidney disease, heart failure, right atrial pressure, Venous Excess Ultrasound

## Abstract

Heart failure (HF) is a major cause of hospitalization, often leading to acute kidney injury (AKI) due to venous congestion. The Venous Excess Ultrasound (VExUS) score, introduced by Beaubin-Souligny, is a bedside tool for assessing congestion severity and guiding decongestive therapy. VExUS has demonstrated prognostic value in predicting AKI, HF readmission, and mortality. Indeed, guiding decongestive therapy through the VExUS score has been shown to significantly improve the likelihood of achieving faster decongestion. **Objectives**: This review aims to discuss the potential role of VExUS and analyze the recent findings about its relevance in guiding decongestive therapy in patients with acute decompensated HF. **Methods**: A comprehensive literature review was conducted, which identified journal articles focused on VExUS and manual reviews of relevant peer-reviewed journals. **Conclusions**: VExUS is a promising tool for evaluating venous congestion in cardiorenal patients, thereby improving fluid and diuretic management. It provides real-time, non-invasive monitoring that enhances clinical decision-making. However, its accuracy depends on operator expertise, and further research is needed to validate its application across different patient populations.

## 1. Introduction

Cardiorenal syndromes represent a range of conditions characterized by concurrent heart and kidney dysfunction, which is influenced by an interplay of neurohormonal, inflammatory, and hemodynamic disturbances [1]. The management of these patients is challenging due to gaps in understanding their underlying pathophysiology, the lack of objective diagnostic tools available at the bedside, and the presence of individual biases.

Patients with chronic kidney disease (CKD) experience a diminished capacity to effectively remove excess dietary sodium, resulting in fluid retention, which may worsen coexisting heart failure (HF) [1]. Similarly, chronic HF can lead to either acute or chronic renal impairment, primarily due to the retrograde transmission of right atrial pressure (RAP) to the renal veins and interstitial space, a condition referred to as congestive nephropathy [2]. These interrelated mechanisms led to the creation of a classification system proposed by Ronco and colleagues [3], which distinguishes between cardiorenal syndromes—acute (type 1) or chronic (type 2) cardiac dysfunction resulting in renal impairment—and reno-cardiac syndromes, in which acute kidney injury (AKI) (type 3) or chronic kidney disease (type 4) precipitates cardiac dysfunction. Through the assistance of these patients, it has been proven that achieving complete decongestion, even at the cost of worsening kidney function, is linked to better short- and long-term outcomes, a situation known as pseudo-worsening renal function (pseudo-WRF) [4]. However, excessive fluid removal can lead to renal hypoperfusion and subsequent tubular damage. The management of cardiorenal syndrome is largely supportive, addressing both organ systems simultaneously. Common treatments used include diuretics, renin-angiotensin inhibitors, and, in some cases, renal replacement therapy. However, traditional therapies do not specifically target pathophysiology, underscoring the need for improved diagnostic and therapeutic approaches.

Traditional physical examination methods often lack the sensitivity and specificity required to detect residual venous congestion and may present reproducibility challenges, even among experienced clinicians. Signs, such as peripheral edema, jugular distention, and rales, can be present but may not reflect the full extent of congestion or the early stages of cardiorenal dysfunction. Additionally, the complexity of assessing renal function in cardiorenal patients is a challenge; traditional markers such as creatinine may not accurately reflect the real-time status of renal function, particularly in fluid overload or fluctuating hemodynamics. The presence of pseudo-WRF due to aggressive diuretic therapy or the risk of renal hypoperfusion with inappropriate treatment, further complicates its understanding [4]. This makes it crucial to explore new diagnostic tools which can enhance the precision of diagnosing and managing cardiorenal syndrome. Point-of-care ultrasound (POCUS) offers a valuable complement to these conventional assessment techniques, enhancing their accuracy in several ways [5]. In this context, multimodal assessment of congestion has been positioned as the standard of care to prepare individualized decongestive strategies when cardiorenal syndrome and/or congestive nephropathy are present [6].

In 2020, Beaubien-Souligny [7] introduced the VExUS score, a multi-parameter tool designed to assess the severity of congestion in HF and the risk of developing AKI in postoperative cardiac patients. Additionally, VExUS not only provides a non-invasive method for quantifying venous congestion but also enables the real-time monitoring of changes during therapeutic interventions. Despite its potential, the integration of VExUS into routine clinical practice remains limited due to the lack of consensus around its use. This represents a significant gap in current clinical knowledge. This review will explore the potential applications of VExUS and examine recent research highlighting its role in guiding fluid management strategies for cardiorenal patients.

## 2. Venous Excess Ultrasound (VExUS) Score

It involves performing IVC and Doppler evaluation on the hepatic, portal, and renal parenchymal veins. Simultaneous ECG is encouraged for accurate waveform interpretation [8].

○ IVC measurement is the first step for assessing venous congestion. If IVC is greater than 2 cm, it could be explained whether it is due to increased stressed venous volume or an increased RAP.
○ The hepatic vein shows two positive retrograde waves (A and V waves) and two negative antegrade waves (S- and D-waves), indicating blood movement away and toward the heart, respectively. Without congestion, the S-wave is greater than D-wave, reflecting normal conditions (S > D). In mild congestion, the amplitude of the S-wave is lower than D-wave (S < D). In severe congestion, the S-wave is reversed, positioning itself above the baseline, leaving only the D-wave below the baseline. Severe tricuspid regurgitation will usually result in a reverse S-wave with retrograde flow; in this scenario, portal flow is the preferred sonographic marker to track volume [9].
○ Normal portal vein (PV) flow has no pulsatility. It becomes pulsatile with congestion. PV Doppler shows a high degree of agreement with intrarenal venous flow (IRVF) and displays excellent reproducibility even among non-expert sonographers [10]. The correct acquisition should be performed during and toward the end of the expiratory pause to avoid respiratory variation. Regarding drawbacks, in patients with advanced cirrhosis, PV is not reliable. Also, increased PV pulsatility has been described in healthy subjects with a low body mass index [11].
○ About IRVF, normal flow is continuous. In the presence of venous congestion, IRVF becomes interrupted. Increased IRVF pulsatility is linked to diuretic resistance and adverse clinical outcomes [12]. In patients with pulmonary hypertension, interrupted renal flow is associated with right heart dysfunction and independently predicts morbidity and mortality [13]. Focused cardiac ultrasound is suggested when altered IRVF is encountered. Sometimes, IRVF evaluation is challenging, and its use in advanced renal fibrosis may be less reliable. It needs more validation in the chronic hemodialysis population. Several authors support assessing PV flow instead of IRVF, given the technical difficulties, which can sometimes be time-consuming [14].

VExUS is categorized into four grades based on IVC and the presence of abnormal venous flow patterns:

0. VExUS 0 (no congestion): IVC < 2 cm.
1. VExUS 1 (mild congestion): IVC > 2 cm with any combination of normal or one mildly disturbed venous flow pattern (hepatic, portal, or intrarenal).
2. VExUS 2 (moderate congestion): IVC > 2 cm with at least one severe disturbed venous flow patterns.
3. VExUS 3 (severe congestion): IVC > 2 cm with two or more severely disturbed flow.

**Figure 1:**
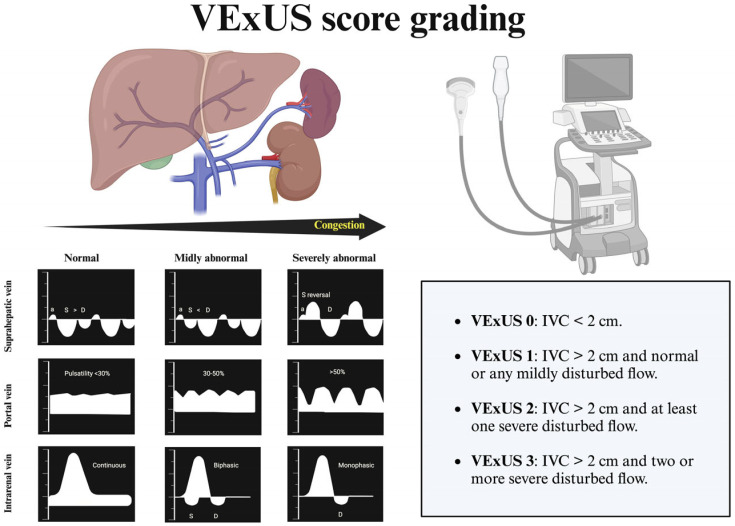
VExUS score grading. Figure created with Biorender. a wave: atrial contraction; S wave: ventricular systole; D wave: ventricular diastole.

The VExUS score demonstrated greater specificity in predicting AKI compared to its individual components [7]. More recently, femoral vein Doppler has been suggested as a simpler site for assessing congestion, showing a moderate correlation with the VExUS score [14]. Figure 1 shows the VExUS grading score [15].

## 3. VExUS Usefulness

### 3.1. Acute Kidney Injury (AKI)

According to the Kidney Disease Improving Global Outcomes (KDIGO) criteria [16], AKI is defined by any of the following: a serum creatinine increase of ≥0.3 mg/dL within 48 h, a 50% or greater rise in serum creatinine from baseline, or a urine output of less than 0.5 mL/kg/hour for a period of 6 h. The management of AKI in the emergency department can be challenging due to the uncertainty surrounding its etiology, which may include hypovolemia, cardiorenal syndrome, systemic vasodilation, and renal factors [17]. Hypovolemia is responsible for approximately 50% of AKI cases in the emergency setting, with treatment primarily focusing on volume resuscitation. In contrast, AKI associated with cardiorenal syndrome is typically linked to volume overload and is managed through volume removal. Thus, accurately identifying the underlying cause of AKI is crucial for determining the most appropriate therapeutic approach.

**Figure 2:**
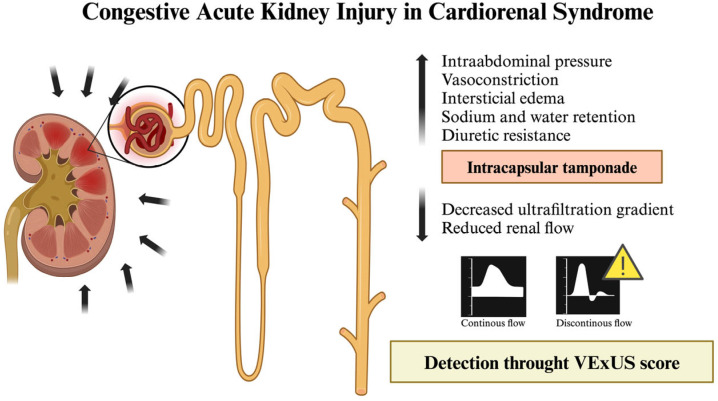
Illustration of AKI with secondary HF due to volume overload. Figure created with Biorender.

Recent studies indicate that the VExUS score demonstrates good diagnostic accuracy for cardiorenal AKI and moderate accuracy for hypovolemic AKI; however, it is not effective in distinguishing renal and systemic vasodilation subtypes [18]. This highlights why the VExUS score should not be exclusively relied on to determine the underlying cause of AKI. The traditional role of hypoperfusion as the main driver of cardiorenal syndrome is increasingly being questioned. In cases of decompensated HF, the involvement of splanchnic veins is common, with a progressive increase in intra-abdominal pressure—a phenomenon known as renal intracapsular tamponade—that contributes to AKI due to volume overload [19,20] (Figure 2). Mullens et al. [19] carried out a prospective observational study, including 40 patients with acute HF who were admitted to the intensive care unit. Their findings demonstrated that elevated central venous pressure, rather than reduced cardiac output, was the primary driver of AKI in these patients, highlighting the critical role of venous congestion in worsening renal function. Additionally, Rubio et al. [20] conducted a prospective observational study involving 56 patients with AHF, focusing on the impact of elevated intra-abdominal pressure on diuretic response and clinical outcomes. Their results showed that patients with higher intraabdominal pressure exhibited poorer diuretic response, higher mortality rates, and increased rehospitalization rates, reinforcing the importance of assessing intra-abdominal congestion in HF management.

Although venous congestion plays a central role in renal dysfunction in HF patients, a significant reduction in cardiac output can further impair renal perfusion. As a result, estimating cardiac output is a crucial component of a thorough hemodynamic assessment in individuals with heart failure and oliguria or AKI. In the initial study by Beaubien-Souligny [7], which included 145 patients undergoing cardiac surgery, a positive likelihood ratio of 6.37 was reported for the development of cardiorenal AKI in individuals with severe venous congestion. A more recent prospective study included 77 patients with acute coronary syndrome, and identified a correlation between VExUS grading and the occurrence of AKI, with higher VExUS grades associated with a significantly increased risk of renal impairment [21]. Additionally, recent evidence from the latest prospective study by Sovetova et al. demonstrated that among 100 patients with a VExUS grade 3, there was a higher risk of developing AKI (with an incidence of 47%), as well as diuretic resistance with a reduced natriuretic response [22]. These findings align with previous studies, reinforcing the prognostic value of VExUS. Moreover, patients with VExUS 3 required higher doses of ionotropic support and had a higher mortality rate compared to those with lower VExUS grades [22].

### 3.2. Diuretic Resistance and Diuretic Theraphy

There is currently no consensus on the accurate definition of diuretic resistance. It is generally described as the incompetence to achieve sufficient fluid and sodium excretion to alleviate volume overload, despite increasing doses of loop diuretics [23]. Typically, oral and ambulatory doses of ≥80 mg of furosemide or the combination of different oral diuretic therapies to achieve euvolemia are considered the threshold for diagnosing diuretic resistance. Other definitions include the need to double the initial dose of furosemide when one of two conditions is met: spot natriuresis of less than 50 mmol/L and/or a 6-h urine output below 100 mL/h [24].

There is a potential role for VExUS in predicting diuretic response during HF hospitalizations. Enough evidence suggests that serum creatinine levels are unreliable markers for assessing renal function or the success of diuretic therapy during AKI [25]. VExUS may play a pivotal role in identifying early signs of diuretic resistance, enabling timely adjustments to therapy, and potentially improving fluid management outcomes [26]. This idea is supported by several studies showing that effective diuretic therapy leads to improvements in the VExUS grade and intrarenal blood flow patterns [27,28]. Alterations in intra-abdominal flows can be modified with decongestion. In HF patients, the restoration of IRVF was linked to better prognosis, as shown by Yamamoto et al., whose study identified discontinuous renal flow as a predictor of cardiovascular death and readmission for HF [29]. Additionally, in patients with HF or pulmonary hypertension and acute cardiorenal syndrome, diuretic therapy resulted in the normalization of portal flow, coinciding with the resolution of AKI [30]. Despite the potential for modest reductions in renal blood flow, decongestion should still be pursued, a strategy known as “permissive AKI” [31]. For CKD patients, Tonelli et al. carried out a prospective observational study of 41 patients with end-stage kidney disease who required intermittent hemodialysis or ultrafiltration. Their results revealed a high prevalence of venous congestion in patients undergoing hemodialysis and determined that changes in portal flow were more sensitive than changes in IVC in monitoring real-time decongestion during volume removal in hemodialysis patients [32]. However, evidence in this population remains limited, and further research is needed.

A recent randomized clinical trial led by Islas et al. compared diuretic treatment guided by VExUS with standard clinical care in patients with cardiorenal syndrome type 1. A total of 140 patients were randomized 1:1. The study concluded that while VExUS-guided decongestion did not lead to improved kidney function recovery, it did significantly increase the likelihood of achieving decongestion more than two times faster [33]. Traditionally, the recovery of renal function, as indicated by serum creatinine levels, has been seen as a positive outcome under the assumption that it reflects functional improvement of the kidneys. However, paradoxically, during diuretic-induced decongestion, an increase in creatinine has been associated with better decongestion and improved cardiorenal survival, challenging this conventional view and suggesting a new paradigm for clinical practice [34]. These findings align with international guidelines, which emphasize that faster decongestion correlates with better cardiorenal outcomes [24].

### 3.3. Right Atrial Pressure (RAP), Right Heart Failure and Pulmonary Hypertension

Elevated right-sided heart pressure is linked to a range of adverse outcomes, particularly among the critically ill [35]. The visual assessment of venous congestion through the observation of the internal jugular vein has long been considered a crucial part of the physical examination; however, it is subject to considerable intra-rater and inter-rater variability. Despite ongoing debates, the most accurate method for measuring venous congestion remains right heart catheterization (RHC) with the direct assessment of RAP, which serves as a reliable indicator of venous hypertension [36,37]. However, RHC is not practical for routine clinical evaluations in diverse care settings due to its cost, difficulty in repetition, and associated side effects. These limitations underscore the need for a more accurate, cost-effective, reproducible, and non-invasive method of assessing venous hypertension. In response to this, the VExUS score represents a novel approach that combines previously identified ultrasound markers that are indicative of elevated RAP.

**Figure 3:**
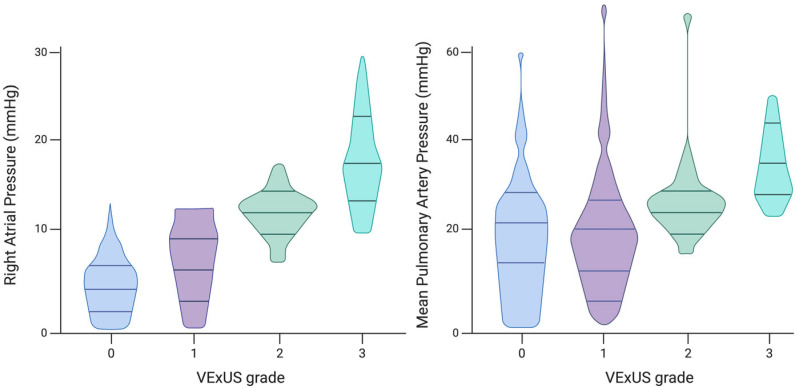
Associations between VExUS grade and intracardiac pressures measured by right heart catheterization. Figure created with Biorender and adjusted Longino A et al. [39].

Preliminary studies have demonstrated strong correlations between VExUS grade and RHC measurements. Longino et al. prospectively assessed the correlation of VExUS with RAP in comparison with IVC diameter. Patients undergoing RHC underwent VExUS examination before the procedure, and ultrasonographers were blinded to RHC outcomes. After adjusting for factors such as age, sex, and common comorbidities, Longino et al. identified a significant positive correlation between RAP and VExUS grade (*p* < 0.001, R^2^ = 0.68) [38] (Figure 3). The VExUS technique showed an excellent area under the curve (AUC) for predicting RAP ≥ 12 mmHg (0.99, 95% CI 0.96–1), outperforming IVC diameter measurement (0.79, 95% CI 0.65–0.92). VExUS demonstrated superior predictive capability for elevated RAP values compared to IVC diameter alone or the collapsibility index. These findings are consistent with a recent study of 124 HF patients, which found that the VExUS score provided more accurate results than IVC characteristics [39].

Furthermore, research by Iida et al. enrolled both hospitalized patients and outpatients and performed a follow-up over a one-year period to determine if IRVF is related to a worse prognosis. The results strongly suggest a correlation between renal congestion and IRVF patterns and worse clinical outcomes, independent of the RAP [12]. In addition, Husain-Syed et al. identified a link between biphasic and monophasic renal blood flow patterns and RAP in patients with pulmonary hypertension, which has also been recognized as a robust predictor of poor clinical outcomes [13]. These findings highlight a significant correlation between VExUS grading and RAP in a wide range of patient populations, suggesting that VExUS could help avoid invasive procedures in critically ill patients and provide valuable guidance for decongestive therapy through ultrasound.

In cases of severe tricuspid regurgitation, often observed in pulmonary hypertension, the portal vein, rather than IRVF or IVC, tracks volume removal during decongestion. Alday and his team [9] prospectively enrolled 42 patients with severe tricuspid regurgitation undergoing decongestive therapy. After volume removal, PV flow improved significantly. A higher proportion of patients displayed an improvement in PV compared to IRVF. PV flow was the only predictor of achieving >5 L of negative fluid balance (AUC 0.83, *p* = 0.001). Despite the persistence of severe tricuspid regurgitation and right ventricular dysfunction, the portal vein pulsatility fraction can improve with volume removal, often reaching values below 50% in most patients. In contrast, improvements in IRVF were only seen in patients who showed a reduction in the severity of tricuspid regurgitation during decongestion [9]. This finding is clinically significant, as there are currently no reliable markers for assessing optimal decongestion in these patients.

### 3.4. Prognosis Predictor

A severe congestion state at admission (identified as VExUS score = 3) has been identified as a strong predictor of mortality and higher HF readmission rates [7,14,22,40]. Moreover, assessments of the portal and renal circulation have shown potential as prognostic markers, suggesting that they may serve as independent predictors of mortality [14,27]. Additionally, several studies involving both outpatient and hospitalized patients with chronic and acute HF have demonstrated that the presence of intrarenal biphasic or monophasic blood flow is linked to poor clinical outcomes, including death from cardiovascular causes and rehospitalizations for HF [12,19,41,42]. Below, in Table 1, a review of previous studies, their design, and main findings is displayed.

## 4. Cardiorenal Units, Drawback, and Future

Cardiorenal syndrome is a complex condition that displays significant challenges and requires a comprehensive, multidisciplinary approach, encompassing primary care and specialized fields such as cardiology, nephrology, and internal medicine. The establishment of specialized care units is essential for improving outcomes, particularly for patients with complex cases or those needing advanced interventions, such as ventricular assist devices, inotropic support, or extracorporeal renal replacement therapies. These units would enable more coordinated, holistic patient care. Despite clinical guidelines recommending such units, their implementation is limited in Spain. Only 10% of HF units have specialized programs for cardiorenal syndrome, and just 30% have collaborative protocols between cardiology and nephrology [43].

### 4.1. Strengths and Drawbacks

Like any diagnostic tool, VExUS has its limitations and must be evaluated individually [44]. For instance, a patient with hepatic cirrhosis and concurrent gastrointestinal bleeding may exhibit portal vein pulsatility despite having intravascular hypotension [45]. Similarly, severe tricuspid regurgitation produces a retrograde flow during systole in the hepatic vein Doppler, even if cardiac filling pressures are low [46]. Special care should be taken with patients who have chronic pulmonary hypertension and high VExUS scores, as aggressive fluid removal could compromise their cardiac output, which may rely on high preload [15]. These example patients might be classified as having moderate venous congestion, underscoring the importance of understanding patient-specific physiology [47]. In terms of sensitivity and specificity, VExUS is promising, but further research is still required. Regarding reproducibility, it is one of the major advantages as it could be performed at different times, but operator dependence can vary based on the clinician’s skill level. In conclusion, using VExUS without a thorough grasp of its technical limitations can lead to clinical misjudgments.

### 4.2. Novel Biomarkers in Cardiorenal Syndrome

The femoral vein, especially the right femoral vein, is an extension of the IVC with a relatively direct path, offering a valuable window to assess IVC and right atrial dynamics. Denault et al. proposed that femoral vein Doppler, a simpler alternative to the VExUS score, could also be useful for evaluating right ventricular overload and signs of venous congestion [48]. Recent studies have supported this strong correlation and introduced a simplified approach, though further validation and investigation are needed [49].

In addition to ultrasound assessment, there is increasing evidence supporting the validation of cardiorenal biomarkers, such as microRNAs, which can assist clinicians in overcoming therapeutic limitations and developing new strategies for managing complex cardiorenal conditions [50].

### 4.3. Future Research

Looking ahead, future research should aim to conduct clinical trials, including VExUS scores in advanced HF and CKD, among frail patients who better represent the typical clinical practice. Specifically, the integration of VExUS-guided decongestive diuretic therapy could offer personalized management, minimizing hypoperfusion or unmasked residual congestion. Additionally, studies should focus on standardizing its protocols to minimize operator variability and explore the integration of VExUS with other advanced diagnostic tools for a more comprehensive approach to cardiorenal syndrome. Ultimately, the development of more robust, evidence-based guidelines for VExUS will play a crucial role in improving patient care and outcomes.

## 5. Conclusions

VExUS represents a promising tool for cardiorenal syndrome patients, offering real-time, non-invasive monitoring of fluid overload and renal function. It has demonstrated strong prognostic value, correlating with mortality risk, AKI, and diuretic resistance, and guiding more efficient decongestion strategies. Despite its potential, VExUS requires operator expertise and careful interpretation to avoid clinical misjudgments. Further research, including interventional studies, is needed to validate its use across diverse patient populations and compare it to conventional methods. The integration of VExUS into multidisciplinary units could improve patient outcomes by enabling more tailored therapeutic approaches.

## Figures and Tables

**Table 1:** Review of previous studies regarding VExUS and cardiorenal field.

First Author, Year (Ref.)	Study Design	Outcomes and Results
Torres, 2023 [14].	Prospective, observational	Intrarenal monophasic flow, portal pulsatility > 50%, and VExUS grade 3 predicted mortality.
Islas, 2023 [33].	Randomized clinical trial	VExUS-guided decongestion improved the odds of achieving decongestion.
Espriella, 2023 [27].	Prospective, observational	Discontinuous renal flow at 30 days post-discharged was related to adverse events.
Yamamoto, 2021 [29].	Prospective, observational	Discontinuous renal flow was a predictor of cardiovascular death and readmission for HF.
Sovetova, 2024 [22].	Prospective, observational	VExUS grade 3: higher incidence of AKI, decreased natriuretic response, need of ionotropic support, and higher mortality.
Beaubien-Souligny, 2024 [40].	Prospective, observational	VExUS grade was associated with higher mortality in critically ill patients with AKI.

## Abbreviations

The following abbreviations are used in this manuscript:

AKI	Acute Kidney Injury
CKD	Chronic Kidney Disease
HF	Heart Failure
IRVF	Intrarenal Venous Flow
IVC	Inferior Vena Cava
PV	Portal vein
RAP	Right Atrial Pressure
RHC	Right Heart Catheterization
VExUS	Venous Excess Ultrasound
